# Dietary diversity contributes to delay biological aging

**DOI:** 10.3389/fmed.2024.1463569

**Published:** 2024-10-09

**Authors:** Wen Liao, Meng-ying Li

**Affiliations:** ^1^Department of Nephrology, Pingxiang People’s Hospital, Pingxiang, China; ^2^Department of Haematology, Jiujiang City Key Laboratory of Cell Therapy, Jiujiang No. 1 People’s Hospital, Jiujiang, China

**Keywords:** dietary diversity score, biological aging, NHANES, mediation analysis, glutamyltransferase

## Abstract

**Aims:**

As aging is a major risk factor for chronic diseases, strategies to promote healthy aging are essential. Dietary diversity has been reported to be beneficial for human health, however, the role in the biological aging process remains underexplored. Our aim was to analyse the potential link between diet diversity and aging.

**Methods:**

Twenty-two thousand six hundred participants from the National Health and Nutrition Examination Survey (NHANES) were included in this study. Dietary diversity was assessed by the dietary diversity score (DDS), which aggregated data on participants’ self-reported dietary categories for the 5 major food groups (18 subgroups) over 2 rounds. Biological age was determined using the phenotypic age, with the residual between biological age and chronological age, phenotypic age acceleration, representing biological aging advance. Weighted multivariate regressions analysis were used to examine the relationship between DDS and phenotypic age acceleration. Sensitivity, subgroup interaction and mediation analyses were employed for further analysis.

**Results:**

Higher DDS was consistently associated with slower phenotypic age acceleration (*β* < 0, *p* < 0.001). Subgroup analyses revealed that the inverse relationship persisted across categories, with minimal interaction effects. Sensitivity analyses confirmed the robustness of results. The oxidative stress indicator glutamyltransferase partially mediated the relationship between DDS and aging [4.9% (3.6, 6.0%), *p* < 0.001].

**Conclusion:**

Dietary diversity is associated with a slower rate of biological aging, which may be due in part to reduced oxidative stress. These findings underscore the potential of a rich, broad-spectrum diet to promote healthy aging and reduce the burden of age-related diseases.

## Graphical abstract

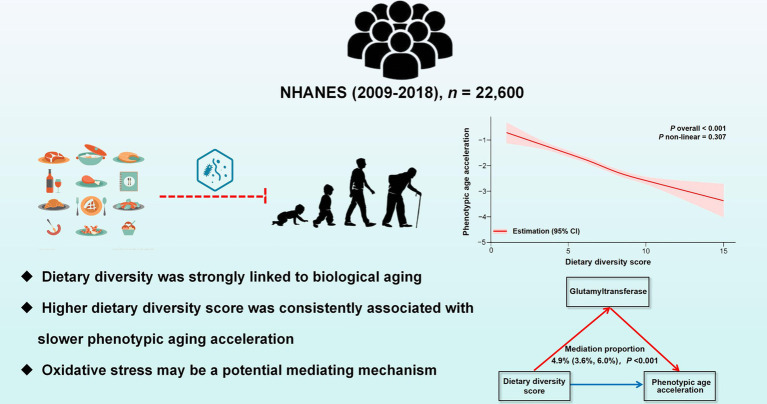

## 1 Introduction

The accelerated aging of the global population constitutes a major public health issue (1). Aging serves as a key risk factor for numerous chronic diseases, imposing considerable economic burdens on society (2). Therefore, it is crucial to develop and implement preventive strategies and interventions that promote healthy aging.

Several factors, including genetic, environmental, and dietary habits, influence the aging process (3–5). Recently, scholars have established a number of dietary quality assessment tools that have been shown to have strong correlations with chronic disease and aging (6). For example, the Mediterranean diet (MED) (7) and the global diet quality score (QDQS) (8) have been associated with reduced mortality and increased healthy life expectancy. Both greater composite dietary antioxidant index (CDAI) (9) and reduced dietary inflammatory indices (DII) (10) have also been shown to be associated with a low risk of biological aging. However, the assessment of individual foods or nutrients does not take into account the effect of an individual’s overall dietary pattern on their health. There is a need to develop integrated and comprehensive dietary aspects to assess the potential relevance to aging.

In fact, dietary diversity is an essential consideration when assessing dietary quality and includes the intake of a variety of foods that contribute to maintaining optimal nutrient absorption. A nutritionally diverse diet, comprising foods rich in macronutrients, micronutrients, antioxidants, and bioactive compounds, is essential (11). Such dietary diversity fosters a healthier gut microbiota, which is pivotal for overall health, encompassing immune function and metabolic processes (12). Moreover, diverse food sources affect multiple aspects of the organism. A diet high in animal protein, especially red meat with elevated levels of methionine and branched-chain amino acids, may promote age-related diseases (13). On the contrary, increasing the intake of whole grains, vegetables, fruits, nuts, and fish could effectively mitigate inflammaging (14, 15). Thus, dietary diversity may better provide a comprehensive overview of overall dietary patterns than the criteria of individual dietary assessment tools (16).

Several dietary indices have been developed to adequately capture dietary variability, including the food variety score (FVS) (17), dietary diversity score (DDS) (18), and healthy food diversity index (HFDI) (19). Of note, the DDS has been shown to be a simple and effective method of estimating the diversity and balance of dietary intake, originally proposed by Kant et al. (20) and its applicability then validated in various clinical trials. DDS is a measure of counts that assesses the intake of foods or food categories, usually several food macrogroups and subgroups, over a specific timeframe. Previous research had also suggested that the DDS is associated with age-related risk factors, including diabetes mellitus (DM) (21), hypertension (22), and cardiovascular diseases (CVD) (23). All of these studies have shown that higher DDS, is associated with a lower risk of chronic disease. Nevertheless, the specific role of DDS in the biological aging process remains underexplored.

Previously, accurate assessment of organismal aging was not an easy task. The aging process is not uniform due to individual differences, resulting in varied rates of aging and differing susceptibilities to mortality and disease among populations (24). Recently, biological age, calculated from several parameters, has been reported to be useful in studying differences in aging rates (25, 26). Representative measurements of biological aging such as phenotypic age and Klemera–Doubal method (KDM) biological age, which are based on composite clinical biomarkers, have been proposed and validated according to previous studies (27). Compared to chronological age, these two novel biological indicators of aging provide a more accurate picture of organismal aging and can be more closely associated with poor prognosis such as all-cause mortality.

In short, the DDS is capable of estimating food richness, while representative novel indicators of biological age can effectively assess the rate of aging. Given that there are gaps in the link between food diversity and aging, we conducted this experiment using these practical evaluation tools. Furthermore, data from the National Health and Nutrition Examination Survey (NHANES), a practical database reflecting the nutritional status of populations in relation to disease (28, 29), was employed in this study.

## 2 Materials and methods

### 2.1 Study population

**Figure 1:**
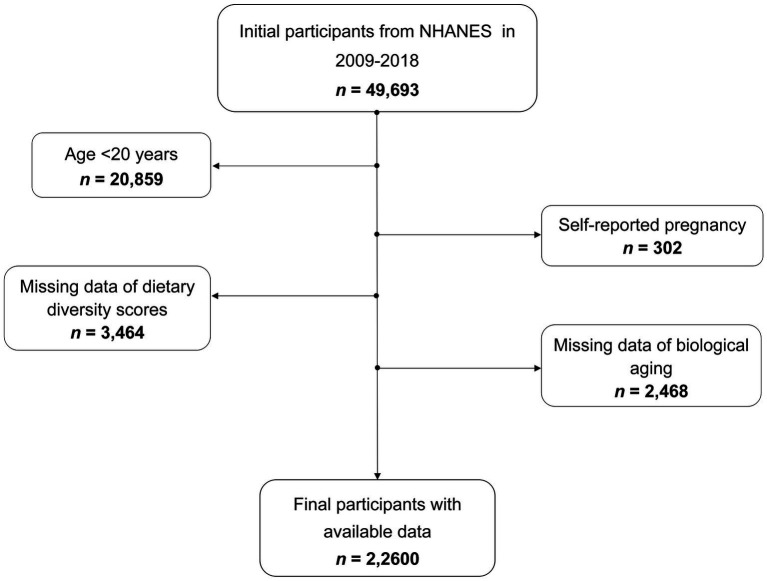
Flow chart for participant selection.

The NHANES database is a comprehensive cross-sectional survey that conducted in the United States, comprised both health interview survey and physical health examination.^1^ Data collection from NHANES participants has received approval from the ethics review board of the National Center for Health Statistics (NCHS). For our study, we integrated data from five NHANES cycles spanning from 2009 to 2018, initially including 49,693 participants. Due to the age <20 years or self-reported pregnancy, 21,161 participants were eliminated. We further excluded 3,464 individuals whose DDS information was not available. Additionally, 2,468 people with no biological age data were ruled out. After applying these exclusion criteria, the final study population comprised 22,600 participants (11,173 males and 11,427 females), flow chart for participant selection was presented in Figure 1.

### 2.2 Data collection

#### 2.2.1 Exposure variable: DDS

The DDS in this study was constructed based on the number of dietary categories in five major food groups (18 subgroups in total), according to the Food and Agriculture Organization of the United Nations food category classification guidelines and similar studies (30, 31). These include grains (whole grains, non-whole/refined grains), vegetables (dark green leafy, vitamin A-rich), fruits (citrus, vitamin A-rich), meat and protein alternatives (red meat, fish and seafood, poultry, organ meat, eggs, legumes and nuts), and dairy products (milks, concentrated dairy products, and solid dairy products). The DDS calculated the total score for all 18 subcategories, ensuring that the same food item was not counted more than once within the same subcategory. Detailed data on the foods consumed by the participants were collected based on two 24-h dietary recalls obtained from NHANES. The DDS was then calculated for each of the 2 days and averaged. A higher DDS is generally indicative of a more varied diet and is associated with a broader intake of essential nutrients. Further details on the methodology employed to derive the DDS can be found in the Supplementary material.

#### 2.2.2 Outcome variables: biological aging

Biological age measurements serve as reliable indicators that more accurately reflect biological aging compared to chronological age. The BioAge R package has developed several validated algorithms to measure biological aging using NHANES data^2^ (32). Two different measures were employed to evaluate biological age: phenotypic age and KDM biological age, derived from an algorithm that estimates mortality risk in a reference population based on biomarkers. By comparing a participant’s biological age with their chronological age, it is possible to appreciate the health status and risk of developing age-related diseases. This measure offers a more precise assessment of an individual’s biological age and risk for age-related diseases compared to chronological age alone, especially phenotypic age (33, 34).

In this study, we obtained biological age assessment variables from the NHANES dataset and used systolic blood pressure, blood creatinine, urea nitrogen, albumin, total cholesterol, glycosylated hemoglobin A1c (HbA1c), percentage of lymphocytes, mean erythrocyte volume, leukocyte count, and alkaline phosphatase as biomarkers of aging phenotype. To assess the rate of aging, phenotypic age acceleration and KDM biological age acceleration were calculated. This was calculated by taking the linear residuals of the two physiological ages (phenotypic age and KDM biological age) and the actual age, respectively. Positive residuals indicate accelerated biological aging and negative residuals indicate decelerated senescence (32–34). A higher biological aging would indicate a greater susceptibility to age-related disease, disability, and death, and vice versa. Given the superior value of phenotypic age, we used phenotypic age acceleration as the primary outcome analysis variable and KDM biological age acceleration as the sensitivity analysis indicator.

#### 2.2.3 Covariates variables

Covariate data were collected using three primary methods: surveys, physical assessments, and laboratory analyses. The surveys gathered information on age, gender, race, education level, poverty income ratio (PIR), health conditions (DM, hypertension, CVD, and cancer), smoking status, alcohol consumption, metabolic equivalent (MET) and dietary intake. Physical assessments included body mass index (BMI). White blood cell count (WBC), neutrophil-lymphocyte ratio (NLR), albumin, total cholesterol, HbA1c, alkaline phosphatase, serum creatinine, glutamyltransferase (GGT) and serum Klotho were laboratory indicators.

Specifically, race were classified into four groups: Mexican American, non-Hispanic white, non-Hispanic black, and others. Education levels were divided into three categories: below high school, high school, and above high school. CVD was identified in participants with a history of congestive heart failure, coronary artery disease, angina, or myocardial infarction. DM was diagnosed based on previous diagnosis, the use of diabetes medication or insulin, fasting blood glucose of ≥7.0 mmol/L, glucose levels of ≥11.1 mmol/L after oral glucose intake, or HbA1c levels of ≥6.5% (35). Hypertension was recognized in individuals with a systolic pressure of ≥130 mmHg, diastolic pressure of ≥90 mmHg, a medical diagnosis of high blood pressure, or the use of blood pressure-lowering medications. Smoking status was divided into never (fewer than 100 cigarettes in a lifetime), former (over 100 cigarettes in a lifetime but not currently smoking), and current (over 100 cigarettes in a lifetime and currently smoking). Alcohol consumption was categorized as follows: never (fewer than 12 drinks in a lifetime), low-to-moderate (up to one drink per day for women and two for men over the past year), and heavy (more than one drink per day for women and two for men over the past year). A physical activity index was calculated as the sum of the estimated MET values for all activities. The individual physical activity index (MET-min/week) was derived by multiplying the MET value by the frequency per week and the activity duration. The participants were divided into three groups based on their MET score: none or <600 (min/week), 600–3,999 (min/week), and ≥4,000 (min/week) (36). The average daily caloric intake was assessed over a two-day period using data from a food consumption survey. Detailed measurement methodologies are available at https://www.cdc.gov/nchs/nhanes/.

### 2.3 Statistical analyses

Statistical analyses were conducted in accordance with the guidelines for the analysis of data from the NHANES, and weighted were applied. The baseline characteristics of the study participants were grouped according to the distribution of the DDS quartiles or the distribution of the phenotypic age acceleration tertiles. Continuous variables were presented as adjusted means ± standard deviation or as medians with interquartile ranges. Categorical variables were described using unweighted numbers (weighted percentages). The statistical significance of the observed differences between the groups was evaluated using the chi-square test and one-way analysis of variance (ANOVA). Weighted multiple linear regression was employed to estimate regression coefficients (*β*) and 95% confidence intervals (95% CI) for DDS in relation to phenotypic age acceleration across three statistical models. Model 1 did not include any confounding variables. Model 2 was adjusted for demographic factors, including age, gender, race, PIR and educational level. Model 3 further incorporated adjustments for CVD, hypertension, DM, cancer, BMI, smoking status, alcohol consumption, dietary energy and MET. Weighted logistic regression models were also employed with biological age acceleration greater than zero as the outcome variable.

Furthermore, subgroup and interaction analyses were conducted to explore the potential interactive effects of various covariates, which were pre-identified as possible effect modifiers. Restricted cubic spline (RCS) regression with four knots was employed to examine the potential non-linear trends between DDS and phenotypic age acceleration. To ensure the robustness of our findings, four sensitivity analyses were also performed using alternative exposure or outcome variables and adjusted datasets. All statistical procedures were carried out using the R software, version 4.3.2 (https://www.R-project.org; R Foundation, Austria). A *p*-value of less than 0.05 was considered statistically significant.

## 3 Results

### 3.1 Participant characteristics

The study covered 22,600 participants. The weighted baseline characteristics of the study population by phenotypic age acceleration tertiles were shown in Table 1. The participants had an average age of 47.8 ± 16.9 years, of whom 11,173 (49.3%) were male. The mean DDS was 6.7 ± 1.9. As phenotypic age acceleration increased, there was a concomitant decrease in BMI, diet energy, WBC, NLR, HbA1c, alkaline phosphatase, serum creatinine, and GGT. Furthermore, the proportion of males, current smokers, and heavy drinkers significantly increased with higher tertiles. Additionally, with increasing phenotypic age acceleration, the prevalence of CVD, hypertension, and DM also increased.

**Table 1:** Characteristics of participants by tertiles of the phenotypic aging acceleration, weighted.

	Total *n* = 22,600	Phenotypic age acceleration			
		Tertile 1 (<−4.40) *n* = 7,534	Tertile 2 (−4.40 to −0.65) *n* = 7,533	Tertile 3 (*≥*−0.65) *n* **=** 7,533	*p*-value
Age, years	47.8 ± 16.9	48.7 ± 16.0	46.0 ± 17.0	48.9 ± 17.4	<0.001
KDM biological age	45.4 ± 17.3	43.4 ± 15.5	42.9 ± 16.4	50.5 ± 19.1	<0.001
KDM biological age acceleration	−2.7 (−7.1, 1.7)	−5.4 (−9.5, −1.1)	−3.0 (−6.9, 0.7)	0.9 (−3.7, 5.8)	<0.001
Phenotypic age	45.3 ± 17.6	41.7 ± 15.9	43.4 ± 17.1	51.6 ± 18.2	<0.001
Male, *n* (%)	11,173 (49.3)	2,390 (32.6)	3,925 (53.6)	4,858 (63.4)	<0.001
Race, *n* (%)					<0.001
Mexican American	3,346 (8.5)	1,232 (8.8)	1,136 (8.6)	978 (8.0)	
Non-Hispanic White	9,388 (67.5)	2,898 (67.8)	3,273 (68.5)	3,217 (66.0)	
Non-Hispanic Black	4,614 (10.3)	1,205 (7.8)	1,457 (9.7)	1,952 (13.9)	
Others	5,252 (13.8)	2,199 (15.7)	1,667 (13.2)	1,386 (13.9)	
Poverty income ratio	3.0 ± 1.6	3.2 ± 1.6	3.0 ± 1.6	2.7 ± 1.6	<0.001
Education levels, *n* (%)					<0.001
Less than high school	2,107 (4.7)	748 (4.9)	640 (4.2)	719 (5.2)	
High school	8,056 (32.4)	2,283 (26.4)	2,649 (32.0)	3,124 (39.6)	
More than high school	12,437 (62.9)	4,503 (68.8)	4,244 (63.8)	3,690 (55.2)	
Cardiovascular disease, *n* (%)	2,373 (8.5)	437 (5.0)	656 (7.4)	1,280 (13.8)	<0.001
Hypertension, *n* (%)	8,186 (32.1)	2,043 (24.7)	2,485 (29.4)	3,658 (43.6)	<0.001
Diabetes mellitus, *n* (%)	4,122 (13.8)	663 (6.4)	1,059 (10.0)	2,400 (26.6)	<0.001
Cancer, *n* (%)	2,143 (10.5)	637 (10.7)	701 (10.0)	805 (10.7)	0.098
Body mass index, kg/m^2^	29.1 ± 6.8	27.0 ± 5.6	29.1 ± 6.3	31.7 ± 7.8	<0.001
Diet energy, kcal/day	2,095 ± 799	1,990 ± 725	2,141 ± 789	2,163 ± 876	<0.001
Smoking status, *n* (%)					<0.001
No	12,687 (56.1)	5,032 (64.4)	4,296 (57.1)	3,359 (45.5)	
Former	5,432 (25.1)	1,687 (25.1)	1,748 (24.0)	1,997 (26.5)	
Current	4,481 (18.8)	815 (10.5)	1,489 (18.9)	2,177 (28.0)	
Alcohol consumption, *n* (%)					<0.001
No	6,837 (23.5)	2,545 (25.1)	2,083 (20.9)	2,209 (24.7)	
Low-to-moderate	14,074 (67.5)	4,537 (67.1)	4,859 (69.7)	4,678 (65.5)	
Heavy	1,689 (9.0)	452 (7.8)	591 (9.4)	646 (9.8)	
Metabolic equivalent, min/week					<0.001
<600	8,763 (34.3)	2,920 (34.1)	2,744 (31.5)	3,099 (37.7)	
600–3,999	8,086 (38.5)	2,986 (43.0)	2,698 (38.3)	2,402 (33.7)	
≥4,000	5,751 (27.2)	1,628 (22.9)	2,091 (30.3)	2,032 (28.7)	
WBC, 1,000 cells/uL	7.2 ± 2.0	6.0 ± 1.4	7.1 ± 1.6	8.5 ± 2.3	<0.001
NLR	2.18 ± 1.13	1.78 ± 0.75	2.17 ± 0.94	2.71 ± 1.45	<0.001
Albumin, g/L	42.7 ± 3.3	43.7 ± 2.9	42.8 ± 3.1	41.3 ± 3.4	<0.001
Total cholesterol, mmol/L	4.9 ± 1.0	5.1 ± 1.0	4.9 ± 1.0	4.7 ± 1.0	<0.001
Glycosylated hemoglobin A1c, %	5.6 ± 0.7	5.4 ± 0.4	5.0 ± 0.5	5.9 ± 1.1	<0.001
Alkaline phosphatase, U/L	68.0 ± 21.1	62.6 ± 18.2	67.3 ± 20.1	74.8 ± 23.2	<0.001
Serum creatinine, umol/L	77.3 ± 19.5	67.9 ± 13.5	77.3 ± 15.4	88.0 ± 23.5	<0.001
Glutamyltransferase, U/L	19 (14, 29)	16 (12, 24)	19 (14, 28)	23 (16, 35)	<0.001
Serum Klotho, (pg/mL)	844 ± 291	872 ± 298	832 ± 275	818 ± 293	<0.001
Dietary diversity score	6.7 ± 1.9	7.1 ± 1.9	6.7 ± 1.9	6.3 ± 1.9	<0.001

*n* (%): unweighted numbers (weighted percentage). KDM, Klemera–Doubal method; WBC, white blood cell count, NLR, neutrophil-lymphocyte ratio.

Meanwhile, Supplementary Table S1 presented the basic demographic details and covariates of the study population according to DDS quartiles. We found that the biological age acceleration gradually advanced with rising DDS. Furthermore, several prognostic indicators that usually predict a poor prognosis, such as WBC, NLR and GGT increased significantly, whereas the level of the anti-aging indicator Klotho decreased. This suggested a potential negative correlation between DDS and the biological aging process. In addition, several key characteristics of excluded (≥20 years) versus included subjects are briefly reported in Supplementary Table S2.

### 3.2 Relationship between DDS and biological aging

Weighted linear regression analysis was conducted to examine the association between DDS and phenotypic age acceleration, and the results were summarized in Table 2. In Model 3, after adjusting various confounders, DDS had negative associations with phenotypic age acceleration (*β* = −0.33; 95% CI: −0.36, −0.30; *p* < 0.001). Similar results were observed in categories, DDS interquartile groupings were inversely correlated with phenotypic age acceleration (*β* = −1.58; 95% CI: −1.73, −1.42; *p* for trend <0.001). The situation was similar for the other 2 models.

**Table 2:** The multiple linear regression analysis for the association between dietary diversity score and phenotypic age acceleration, weighted.

Dietary diversity score	Model 1		Model 2		Model 3	
	*β* (95% CI)	*p*-value	*β* (95% CI)	*p*-value	*β* (95% CI)	*p*-value
Continuous	−0.39 (−0.42, −0.36)	<0.001	−0.39 (−0.42, −0.35)	<0.001	−0.33 (−0.36, −0.30)	<0.001
Quintile 1	Reference		Reference		Reference	
Quintile 2	−0.78 (−0.96, −0.61)	<0.001	−0.75 (−0.92, −0.58)	<0.001	−0.60 (−0.76, −0.45)	<0.001
Quintile 3	−1.19 (−1.35, −1.04)	<0.001	−1.19 (−1.33, −1.04)	<0.001	−0.99 (−1.12, −0.84)	<0.001
Quintile 4	−1.95 (−2.11, −1.78)	<0.001	−1.92 (−2.08, −1.76)	<0.001	−1.58 (−1.73, −1.42)	<0.001
*p* for trend		<0.001		<0.001		<0.001

*β* (95% CI): estimate regression coefficients (95% confidence intervals); Model 1:crude model; Model 2: adjusted for age, gender, race, poverty income ratio and educational level; Model 3: adjusted for Model 2 and hypertension, cardiovascular disease, diabetes mellitus, cancer, body mass index, smoking status, alcohol consumption, dietary energy and metabolic equivalent.

We further applied weighted logistic regression, where phenotypic age acceleration greater than 0 indicates accelerated aging (Table 3). In Model 1 (without adjusting for covariates), the continuous variable DDS was significantly inversely associated with accelerated aging (OR: 0.86; 95% CI: 0.83, 0.88; *p* < 0.001). Further adjustment for demographic variables, diseases, and lifestyle factors (Model 3) did not considerably affect the results (OR: 0.84; 95% CI: 0.82, 0.86; *p* < 0.001). Consistent with previous results, participants in the highest quartiles of DDS showed an inverse correlation with risk of accelerated aging in all three models.

**Table 3:** The results of logistic regression analysis for the association between dietary diversity score and accelerated phenotypic age, weighted.

Dietary diversity score	Model 1		Model 2		Model 3	
	OR (95% CI)	*p*-value	OR (95% CI)	*p*-value	OR (95% CI)	*p*-value
Continuous	0.86 (0.83, 0.88)	<0.001	0.84 (0.82, 0.86)	<0.001	0.84 (0.82, 0.86)	<0.001
Quintile 1	Reference		Reference		Reference	
Quintile 2	0.72 (0.64, 0.82)	<0.001	0.71 (0.62, 0.80)	<0.001	0.72 (0.63, 0.82)	<0.001
Quintile 3	0.63 (0.57, 0.70)	<0.001	0.60 (0.55, 0.67)	<0.001	0.61 (0.55, 0.68)	<0.001
Quintile 4	0.45 (0.40, 0.52)	<0.001	0.42 (0.37, 0.48)	<0.001	0.43 (0.38, 0.49)	<0.001
*p* for trend		<0.001		<0.001		<0.001

OR (95% CI): odd ratio (95% confidence intervals); Model 1:crude model; Model 2: adjusted for age, gender, race, poverty income ratio and educational level; Model 3: adjusted for Model 2 and hypertension, cardiovascular disease, diabetes mellitus, cancer, body mass index, smoking status, alcohol consumption, dietary energy and metabolic equivalent.

### 3.3 RCS analysis of the association between DDS and biological aging

**Figure 2:**
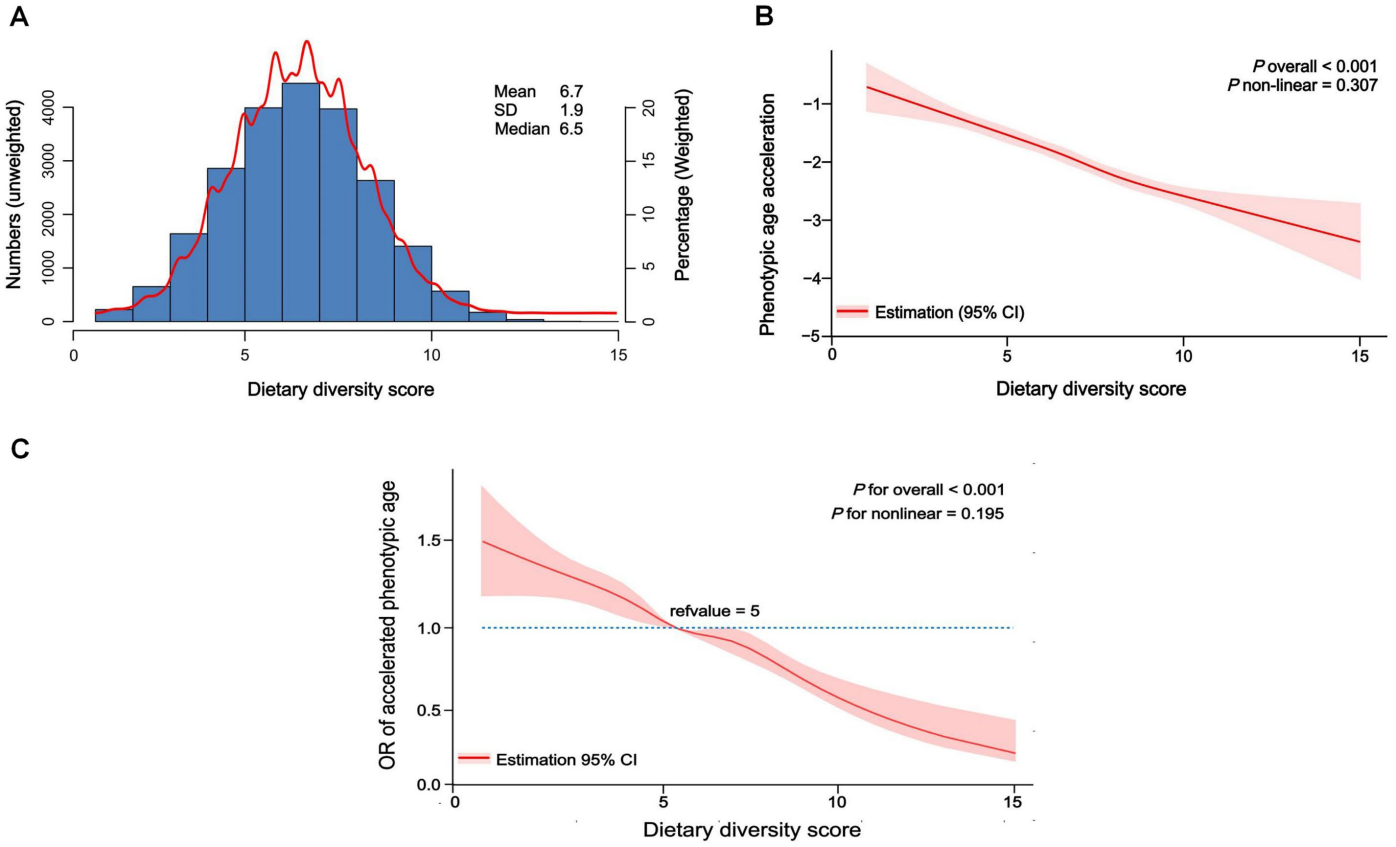
Distribution of dietary diversity score in participants and restricted cubic spline of dietary diversity score and biological aging. (A) Distribution of dietary diversity score in all participants, unweighted. (B) Restricted cubic spline of dietary diversity score and phenotypic age acceleration, weighted adjusted linear regression. (C) Restricted cubic spline of dietary diversity score and accelerated phenotypic aging, weighted adjusted linear regression.

As shown in Figure 2B, the overall relationship between DDS and phenotypic age acceleration was highly significant, implying that DDS had a meaningful effect on phenotypic age acceleration (*p* for overall <0.001). The results also indicated that the relationship between the two was linear, as showed by *p* for non-linear = 0.307. The identical result was noted in DDS with accelerated biological aging (*p* for overall <0.001, *p* for non-linear = 0.195) (Figure 2C). These may imply that more complex models (non-linear) are not necessary for capturing the effect of DDS on the aging outcome.

### 3.4 Subgroup analysis

**Figure 3:**
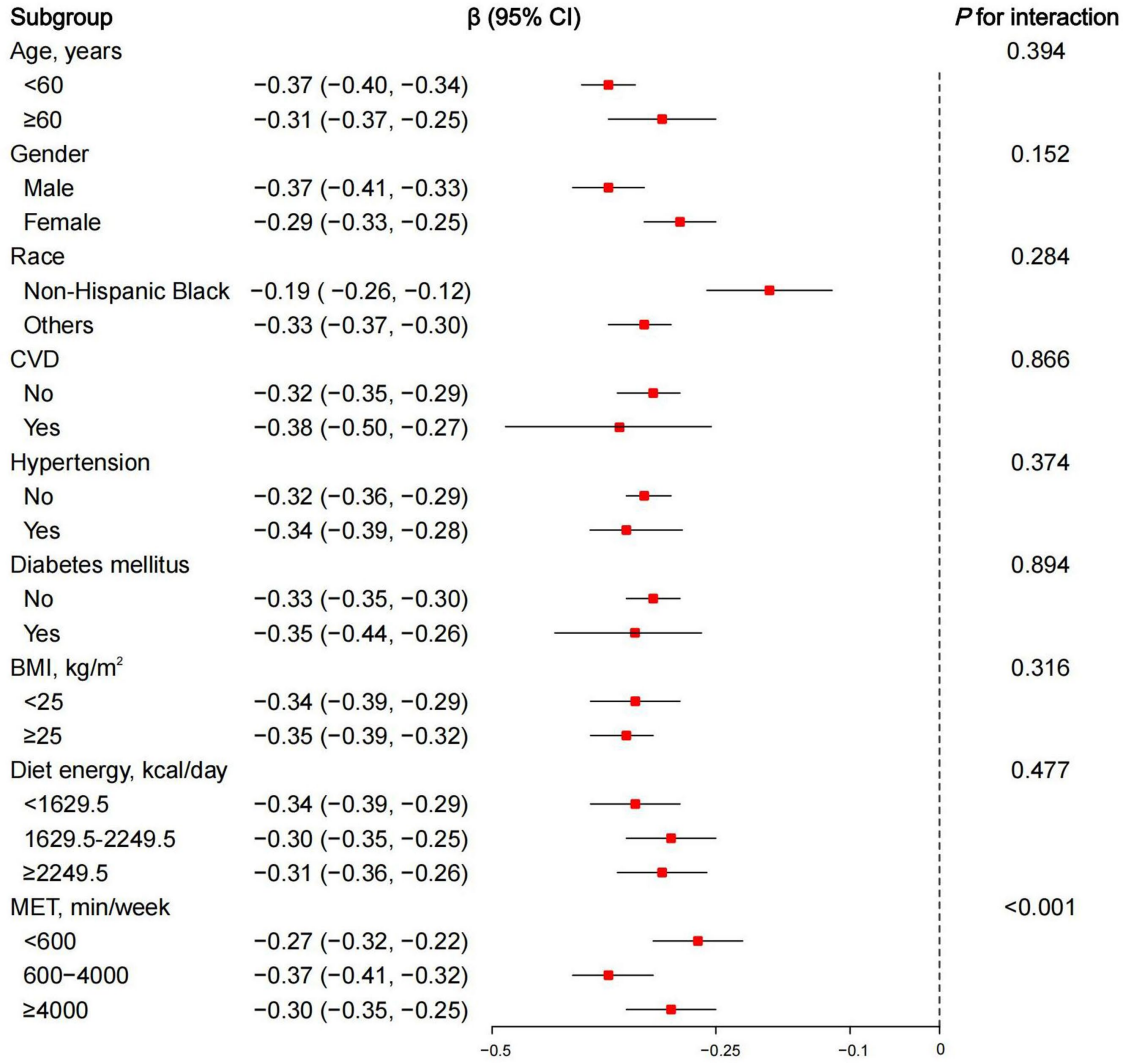
Subgroup analysis of dietary diversity score and phenotypic age acceleration, weighted adjusted linear regression. *β* (95% CI): regression coefficient (95% confidence interval); CVD, cardiovascular diseases; BMI, body mass index; MET, metabolic equivalent.

We conducted a subgroup analysis using linear regression to examine the relationship between DDS and phenotypic age acceleration across various variables, including age, gender, race, CVD, hypertension, DM, BMI, diet energy, and MET (Figure 3). Most subgroups exhibit negative *β* values, indicating a consistent negative effect across all categories. The interaction results suggested that most subgroups do not show significant interaction effects (*p* for interaction >0.05), implying that the effect is generally consistent. Further, consider that age-related biomarkers are gender-specific. We further summarized the correlations between DDS and biological aging-related markers stratified by gender (Supplementary Table S3). The findings indicated that the correlation between DDS and aging exhibited a general consistency across gender.

### 3.5 Sensitivity analysis

The sensitivity analysis results presented in Table 4 demonstrate the robustness of the linear regression between DDS and phenotypic age acceleration, adjusting for different factors. First, we employed the data without multiple imputation for further analysis. Secondly, we used another measure of aging acceleration, calculated by subtracting chronological age from KDM biological age. Next, we then screened out participants without major illnesses, such as CVD, hypertension, DM, or cancer. Finally, we modified the calculation of phenotypic age acceleration by adding C-reactive protein (CRP) to the original basis. In summary, the *β* was consistently negative in all 3 models, suggesting a higher dietary diversity is significantly associated with lower phenotypic age acceleration, regardless of the adjustment methods employed. The results of these data revealed a robust inverse relationship between DDS and biological aging.

**Table 4:** Sensitivity analysis of linear regression between dietary diversity score (continuous) and phenotypic age acceleration, weighted.

Adjustment methods	Model 1		Model 3		Model 3	
	*β* (95% CI)	*p*-value	*β* (95% CI)	*p*-value	*β* (95% CI)	*p*-value
No multiple interpolation	−0.39 (−0.42, −0.36)	<0.001	−0.39 (−0.42, −0.36)	<0.001	−0.33 (−0.36, −0.30)	<0.001
Alternative age acceleration^a^	−0.26 (−0.31, −0.21)	<0.001	−0.13 (−0.18, −0.08)	<0.001	−0.07 (−0.12, −0.01)	0.008
Datasets with non-illnesses^b^	−0.42 (−0.46, −0.39)	<0.001	−0.33 (−0.37, −0.30)	<0.001	−0.32 (−0.35, −0.29)	<0.001
Changed age acceleration^c^	−0.46 (−0.50, −0.42)	<0.001	−0.46 (−0.50, −0.42)	<0.001	−0.38 (−0.42, −0.34)	<0.001

*β* (95% CI): regression coefficient (95% confidence interval).

a Using the Klemera–Doubal method biological age to calculate age acceleration.

b Cardiovascular disease or hypertension or diabetes mellitus or cancer.

c Increase C-reactive protein to calculate aging (*n* = 11,886).

### 3.6 Mediation analyses

**Figure 4:**
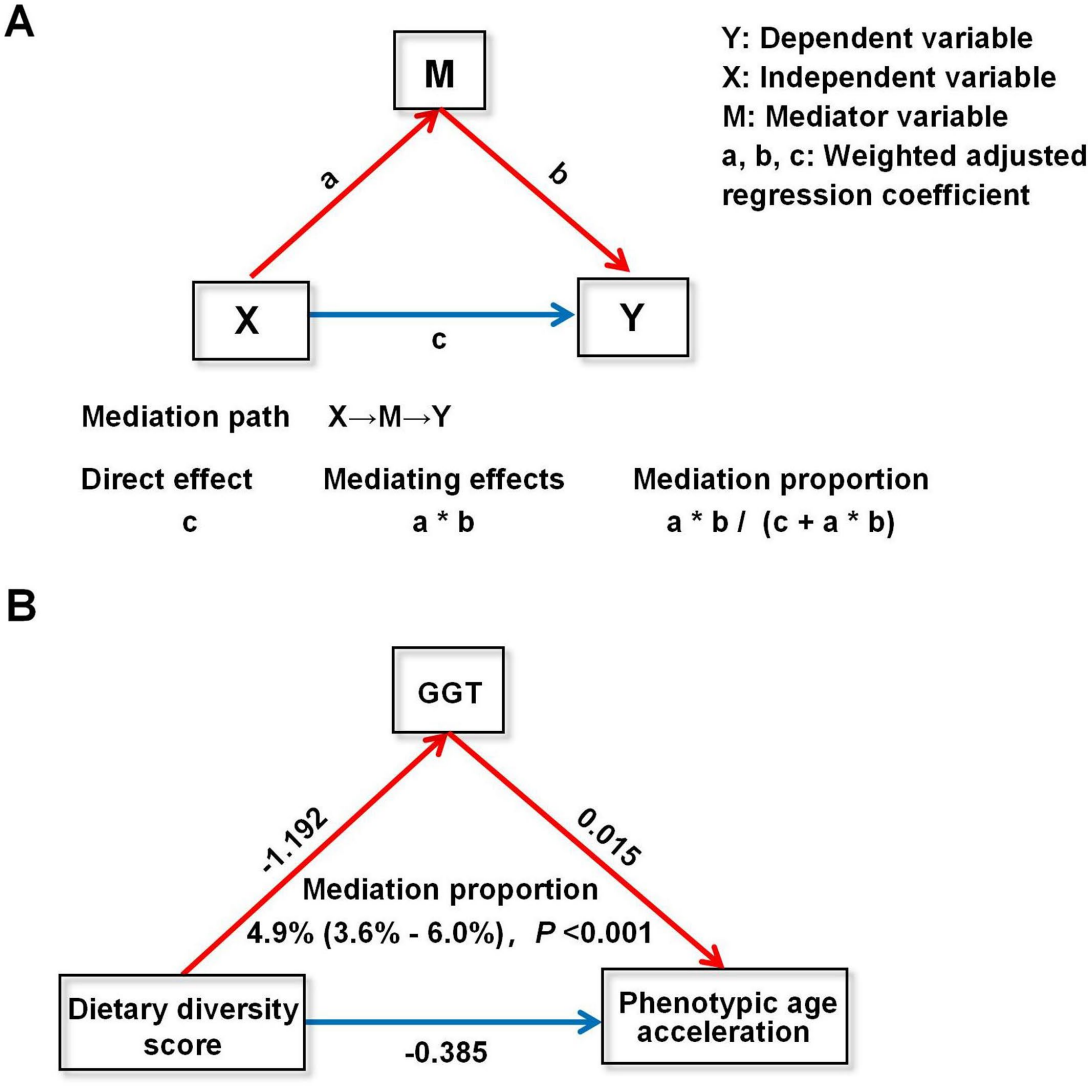
Mediation analysis of dietary diversity score and phenotypic age acceleration. (A) Schematic conceptualization of mediation analysis. (B) Mediation analysis of dietary diversity score and phenotypic age acceleration. GGT, glutamyltransferase.

In addition, a parallel mediation analysis was conducted to assess the potential mediating effect of oxidative stress on the association of food diversity with biological aging (Figure 4A). The oxidative stress indicator GGT had a significant mediating effect on the association of DDS and phenotypic age acceleration, with a mediation ratio of 4.9% (3.6, 6.0%) (Figure 4B).

## 4 Discussion

In this study, DDS was employed to assess the food diversity of participants, while a novel integrated biological algorithm was used to generate quantitative biological aging metrics. The overall results of the study indicated that a higher DDS was associated with slower biological aging. This persistent inverse correlation was observed for both continuous and categorical variables, and the results remained reliable after correcting for multiple confounders and accounting for weighting. Moreover, a series of sensitivity analyses were performed utilising disparate aging acceleration metrics, encompassing the KDM biological age acceleration and aging algorithms covering CRP. The results of these analyses indicate a robust correlation between DDS and aging. In conclusion, the data demonstrate an inverse correlation between DDS and the rate of progression of aging. Promoting dietary diversity may facilitate healthy aging, which has significant implications for public health.

Notably, our study also identified a several features related to aging. Participants with higher DDS exhibited higher serum Klotho, a protein with anti-aging properties (37). In contrast, GGT, an indicator of oxidative stress, WBC and NLR, indicators of inflammation, were significantly lower, and levels of albumin, another potential indicator of the anti-inflammatory capacity of the response machine, were higher. It is noteworthy that the baseline data in Table 1 indicate that while a higher DDS was associated with a higher calorie intake, it was also associated with a lower BMI. A suitable explanation, similar studies in the past have demonstrated that populations with more diverse diets tend to consume more plant-based foods and less animal-based foods, which may be associated with a decrease in BMI (38). Moreover, a greater consumption of low-energy-density foods, such as fruits and vegetables, has been linked to a reduced risk of obesity and metabolic syndrome (39). Usually, obesity and metabolic syndrome were also recognized as risk factors associated with aging (40). These findings suggest that food diversity may exhibit multiple aspects of benefit in slowing the aging process.

As previously mentioned, food diversity reduces the risk of age-related chronic diseases such as depression (41) and sarcopenia (42), and is also associated with decreased mortality (43). In terms of aging, an increasing accumulation of clinical evidence is emerging to suggest that dietary diversity plays a pivotal role in the promotion of healthy aging, which is in alignment with the findings of our study. For instance, Zhang and Zhao (44) developed a healthy aging score (HAS) utilising data from the China Health and Nutrition Survey, whereby scores were standardised for physical functional limitations, comorbidities, cognitive functioning and psychological distress. The findings indicated that there was an inverse association between the DDS and the HAS (T3 vs. T1: *β* −0.16; 95% CI −0.20, −0.11; *p* for trend <0.001). The results of this study imply that dietary habits may exert an influence on the aging process, and that adherence to a varied diet is associated with healthier aging in older adults. Moreover, data from the Longitudinal Survey of Healthy Longevity indicated that a more diverse diet was associated with superior health outcomes in older adults, with a more pronounced association observed in younger older adults compared to older adults (45). Chalermsri et al. (46) found a correlation between dietary diversity and decreased mortality rates among the elderly, especially those aged 70 years or older and those classified as being underweight. These findings align with our initial conclusion that the richer the dietary variety, the later the phenotypic aging.

Although the exact mechanisms linking food diversity to the reduced aging process remain unclear, several possible explanations have been proposed. First, it is important to note that an important aspect of biological aging is cellular senescence. Oxidative stress and inflammation are key phenotypes of cellular senescence, often referred to as senescence-associated secretory phenotypes (SASP) (47). Studies have shown that a varied diet is usually richer in essential nutrients, antioxidants and anti-inflammatory compounds. This provides a more comprehensive array of vitamins, micronutrients, and phytochemicals to support metabolic health and cellular repair processes (44, 48, 49). Again, this may be a key factor in food diversity to form a healthy diet. As evidenced by our mediation analysis, we found that the oxidative stress indicator GGT played a role in the acceleration of DDS with aging, although the percentage of mediator examples was not particularly high. Nevertheless, the importance of this relationship is clear, as oxidative stress leads to a classical phenotype of cellular senescence. In addition, another important factor contributing to the extensive crosstalk between the gut and other organs is the potential benefits of high dietary diversity. These benefits can be attributed, at least in part, to a more homogeneous microbial diversity of gut bacteria. Reduced microbial diversity is usually indicative of a disrupted gut microbiota, which can lead to congestion of the gut wall, edema, slow colonic transit, and impaired intestinal tight junctions. To increased intestinal permeability, bacterial metabolites cross the intestinal barrier, which in turn elicits an immune response and exacerbates organismal aging and damage (12). In conclusion, dietary diversity can be considered as a potential anti-aging factor as it can influence human health.

There are some unique strengths to our research. It is the first to analyse the relationship between food group quantity and aging. The results for weighted large-sample data can be extended to multi-ethnic and multi-type populations. Some optimized statistical analyses demonstrated the robustness of the results. In addition, mediation analyses explained to some extent the potential associations.

Certain limitations warrant consideration in our study. The cross-sectional nature of the study limits the ability to establish causality. Future longitudinal studies are needed to confirm the causal relationship between dietary diversity and biological aging. Additionally, while our study adjusted for a comprehensive set of covariates, residual confounding cannot be entirely ruled out. Another concern is that the DDS assessment does not take into account an individual’s intake of each food item, which may need to be further optimised, and it may be more convincing to use a diversity assessment that includes a food frequency table.

## 5 Conclusion

In conclusion, the results of our study provide compelling evidence that a higher dietary diversity is significantly associated with a lower acceleration of phenotypic age. These findings support the hypothesis that a diverse diet may play a key role in slowing the biological aging process. Nevertheless, due to the significant limitation of cross-sectional observational studies in establishing causal relationships, further prospective interventional experiments are required to validate whether dietary diversity can indeed slow the rate of organismal aging. Moreover, further research is required to elucidate the underlying mechanisms.

## Data Availability

Publicly available datasets were analyzed in this study. This data can be found here: https://wwwn.cdc.gov/nchs/nhanes/.

## Footnotes

^1^ https://www.cdc.gov/nchs/nhanes/

^2^ http://github.com/dayoonkwon/BioAge
