# Supplementary material for: Dietary diversity contributes to delay biological aging
Source: Front Med (Lausanne). 2024 Oct 9;11:1463569. doi: 10.3389/fmed.2024.1463569 (PMC11496103; doi:10.3389/fmed.2024.1463569)
Supplement: Supplementary file 1 [file Table_1.DOC]

**Supplementary Table 1 Characteristics of participants by quartiles of dietary diversity score, weighted**

|  | **Dietary diversity score** | | | |  |
| --- | --- | --- | --- | --- | --- |
|  | **Quartile 1**  **(< 5.5)**  ***n*=** **5,374** | **Quartile 2**  **(5.5 -6.5)**  ***n*=** **5,978** | **Quartile 3**  **(6.5-8.0)**  ***n*=** **6,427** | **Quartile 4**  **(≥8.0)**  ***n*=** **4,821** | ***P*-value** |
| Age, years | 42.4 ± 16.1 | 47.1 ± 16.7 | 49.1 ± 16.7 | 52.2 ± 16.6 | <0.001 |
| KDM biological age | 40.7 ± 16.7 | 44.9 ± 17.1 | 46.5 ± 17.3 | 49.1 ± 16.9 | <0.001 |
| KDM biological age acceleration | -2.2 (-6.4, 2.5) | -2.6 (-7.0, 1.9) | -3.0 (-7.2, 1.5) | -3.4 (-7.7, 1.1) | <0.001 |
| Phenotypic age | 41.0 ± 17.0 | 44.9 ± 17.5 | 46.3 ± 17.62 | 48.7 ± 17.3 | <0.001 |
| Phenotypic age acceleration | -1.8 (-4.4, 1.2) | -2.6 (-5.2, 0.3) | -3.1 (-5.8, -0.1) | -3.8 (-6.3, -0.9) | <0.001 |
| Male, *n* (%) | 2,915 (54.2) | 2,960 (48.8) | 3,096 (48.6) | 2,202 (45.8) | <0.001 |
| Race*, n* (%)  Mexican American  Non-Hispanic White  Non-Hispanic Black  Others | 976 (8.8)  2,773 (63.1)  1,802 (14.1)  1,519 (14.0) | 672 (9.2)  1,706 (65.4)  904 (10.8)  1,000 (14.6) | 1,028 (8.8)  2,678 (68.8)  1,174 (8.8)  1,547 (13.5) | 670 (7.0)  2,231 (73.2)  734 (6.8)  1,186 (13.1) | <0.001 |
| Poverty income ratio | 2.5 ± 1.6 | 2.9 ± 1.6 | 3.1 ± 1.6 | 3.5 ± 1.5 | <0.001 |
| Education levels, *n* (%)  Less than high school  High school  More than high school | 760 (5.9)  3,049 (42.0)  3,261 (52.1) | 458 (5.7)  1,601 (33.9)  2,223 (60.4) | 575 (4.2)  2,127 (29.5)  3,725 (66.3) | 314 (3.2)  1,279 (22.2)  3,228 (74.7) | <0.001 |
| Cardiovascular disease, *n* (%) | 743 (8.1) | 453 (8.4) | 666 (8.8) | 511 (8.8) | 0.698 |
| Hypertension, *n* (%) | 2,427 (30.7) | 1,541 (31.5) | 2,373 (32.8) | 1,845 (33.5) | 0.006 |
| Diabetes mellitus, *n* (%) | 1,215 (12.6) | 790 (13.6) | 1,209 (14.6) | 908 (14.5) | 0.028 |
| Cancer, *n* (%) | 490 (6.9) | 382 (10.5) | 637 (10.9) | 634 (14.5) | <0.001 |
| Body mass index, kg/m^2^ | 29.9 ± 7.4 | 29.3 ± 6.7 | 28.9 ± 6.5 | 28.4 ± 6.5 | <0.001 |
| Diet energy, kcal/day | 1890 ± 795 | 2063 ± 785 | 2170 ± 774 | 2293 ± 785 | <0.001 |
| Smoking status, *n* (%)  No  Former  Current | 2,646 (48.1)  1,014 (19.7)  1,714 (32.2) | 3,272 (54.6)  1,456 (25.2)  1,250 (20.2) | 3,806 (59.4)  1,622 (26.0)  999 (14.6) | 2,963 (61.5)  1,340 (29.3)  518 (9.2) | <0.001 |
| Alcohol consumption, *n* (%)  No  Low-to-moderate  Heavy | 1,607 (23.9)  3,247 (65.0)  520 (11.1) | 1,834 (23.8)  3,698 (67.0)  446 (9.2) | 1,991 (24.2)  3,995 (67.3)  441 (8.4) | 1,405 (21.9)  3,134 (70.8)  282 (7.3) | <0.001 |
| Metabolic equivalent, min/week  <600  600-3999  ≥4000 | 2,899 (36.3)  2,095 (31.9)  2,076 (31.9) | 1,733 (36.1)  1,478 (36.4)  1,071 (27.5) | 2,443 (34.1)  2,431 (40.4)  1,553 (25.5) | 1,688 (30.4)  2,082 (46.6)  1,051 (23.0) | <0.001 |
| WBC, (1000 cells/uL) | 7.5 ± 2.1 | 7.2 ± 2.1 | 7.1 ± 2.0 | 6.8 ± 1.9 | <0.001 |
| Neutrophil-lymphocyte ratio | 2.17 ± 1.10 | 2.18 ± 1.16 | 2.19 ± 1.17 | 2.22 ± 1.13 | 0.022 |
| Albumin, g/L | 42.5 ± 3.5 | 42.6 ± 3.2 | 42.8 ± 3.2 | 42.9 ± 3.1 | 0.015 |
| Total cholesterol, mmol/L | 4.9 ± 1.0 | 4.9 ± 1.05 | 4.9 ± 1.0 | 5.0 ± 1.0 | 0.002 |
| Glycosylated hemoglobin A1c, % | 5.5 ± 0.7 | 5.6 ± 0.7 | 5.6 ± 0.8 | 5.6 ± 0.7 | 0.005 |
| Alkaline phosphatase, U/L | 70.6 ± 21.6 | 68.7 ± 21.4 | 67.0 ± 20.7 | 65.1 ± 20.1 | <0.001 |
| Serum creatinine, umol/L | 77.6 ± 19.7 | 77.5 ± 20.4 | 77.1 ± 19.5 | 76.4 ± 18.5 | 0.033 |
| Glutamyltransferase, U/L | 20 (14, 31) | 19 (14, 30) | 18 (13, 27) | 18 (13, 27) | <0.001 |
| Serum Klotho, (pg/ml) | 838 ± 319 | 853 ± 294 | 855 ± 277 | 858 ± 275 | <0.001 |

*n* (%): Unweighted numbers (weighted percentage); KDM: Klemera-Doubal Method; WBC: White blood cell count
