# Supplementary material for: Dietary diversity contributes to delay biological aging
Source: Front Med (Lausanne). 2024 Oct 9;11:1463569. doi: 10.3389/fmed.2024.1463569 (PMC11496103; doi:10.3389/fmed.2024.1463569)
Supplement: Supplementary file 2 [file Table_2.DOC]

**Supplementary Table 2** Characteristics of excluded (≥20 years) and included participants, weighted.

| **Characteristics** | **Participants excluded**  ***n* =** **6,234** | |  | **Participants included**  ***n* = 22,600** | ***P*-value** |
| --- | --- | --- | --- | --- | --- |
|  | Missing number |  |  |  |  |
| Age, years | - | 48.2 ± 17.2 |  | 47.8 ± 16.9 | 0.099 |
| KDM biological age | *n* = 3,464 | 45.0 ± 16.9 |  | 45.4 ± 17.3 | 0.104 |
| KDM biological age acceleration | *n* = 3,464 | -2.4 (-6.3, 1.4) |  | -2.7 (-7.1, 1.7) | 0.077 |
| Phenotypic age | *n* = 3,464 | 44.9 ± 16.8 |  | 45.3 ± 17.6 | 0.108 |
| Phenotypic age acceleration | *n* = 3,464 | -2.2 (-5.1, 0.5) |  | -2.5 (-5.8, 0.6) | 0.068 |
| Gender  Female, *n* (%)  Male, *n* (%) | - | 2,780 (41.5)  3,454 (58.5) |  | 11,427 (50.7)  11,173 (49.3) | <0.001 |
| DDS | *n* = 3,478 | 6.6±1.9 |  | 6.7±1.9 | 0.097 |
| DDS quartiles  Quartile 1  Quartile 2  Quartile 3  Quartile 4 | *n* = 3,478 | 761 ( 27.6)  751 ( 27.2)  715 ( 25.9)  529 ( 19.3) |  | 5374 ( 25.1)  5978 ( 26.8)  6427 ( 26.9)  4821 ( 21.2) | 0.103 |

*n* (%): unweighted numbers (weighted percentage). KDM: Klemera-Doubal Method; DDS: dietary diversity score
