# Supplementary material for: Dietary diversity contributes to delay biological aging
Source: Front Med (Lausanne). 2024 Oct 9;11:1463569. doi: 10.3389/fmed.2024.1463569 (PMC11496103; doi:10.3389/fmed.2024.1463569)
Supplement: Supplementary file 3 [file Table_3.DOC]

**Supplementary Table 3** Correlation analysis between dietary diversity score and biological aging, stratified by gender, weighted

|  | **Overall** | | **Male** | | **Female** | |
| --- | --- | --- | --- | --- | --- | --- |
| **DDS and phenotypic age acceleration** | **β (95% CI)** | ***P*-value** | **β (95% CI)** | ***P*-value** | **β (95% CI)** | ***P*-value** |
| Continuous | -0.33 (-0.36, -0.30) | <0.001 | -0.37 (-0.41, -0.33) | <0.001 | -0.29 (-0.33, -0.25) | <0.001 |
| Quintile 1 | Reference | | Reference | | Reference | |
| Quintile 2 | -0.60 (-0.76, -0.45) | <0.001 | -0.72 (-0.94, -0.50) | <0.001 | -0.54 (-0.74, -0.32) | <0.001 |
| Quintile 3 | -0.99 (-1.12, -0.84) | <0.001 | -1.11 (-1.30, -1.91) | <0.001 | -0.91 (-1.10, -0.71) | <0.001 |
| Quintile 4 | -1.58 (-1.73, -1.42) | <0.001 | -1.83 (-2.05, -1.61) | <0.001 | -1.43 (-1.64, -1.22) | <0.001 |
| *P* for trend |  | <0.001 | <0.001 | | <0.001 | |
| **DDS and accelerated phenotypic age** | **OR (95% CI)** | ***P*-value** | **OR (95% CI)** | ***P*-value** | **OR (95% CI)** | ***P*-value** |
| Continuous | 0.84 (0.82, 0.86) | <0.001 | 0.83 (0.81, 0.85) | <0.001 | 0.86 (0.83, 0.89) | <0.001 |
| Quintile 1 | Reference |  | Reference |  | Reference |  |
| Quintile 2 | 0.72 (0.63, 0.82) | <0.001 | 0.75 (0.64, 0.88) | 0.001 | 0.68 (0.57, 0.81) | <0.001 |
| Quintile 3 | 0.61 (0.55, 0.68) | <0.001 | 0.59 (0.51, 0.69) | <0.001 | 0.64 (0.55, 0.73) | <0.001 |
| Quintile 4 | 0.43 (0.38, 0.49) | <0.001 | 0.42 (0.36, 0.48) | <0.001 | 0.45 (0.37, 0.55) | <0.001 |
| *P* for trend |  | <0.001 |  | <0.001 |  | <0.001 |

DDS: dietary diversity score; β: estimate regression coefficients; OR: odd ratio; 95%CI: 95% confidence intervals.
