# Supplementary material for: Dietary diversity contributes to delay biological aging
Source: Front Med (Lausanne). 2024 Oct 9;11:1463569. doi: 10.3389/fmed.2024.1463569 (PMC11496103; doi:10.3389/fmed.2024.1463569)
Supplement: Supplementary file 4 [file Data_Sheet_1.DOC]

**Supplementary material**

**Detailed description of the DDS cumulative scoring method：**

In the NHANSE primary data, the food files for each cycle contain a secondary file: the food code description file (DRXFCD_). The food code represents the type of diet consumed by the participant, corresponding to each food item in the Dietary Research Foods and Nutrients Database.

Typically, the first digit is associated with one of the major food categories and the second digit is associated with a more specific subcategory. A more detailed description can be found at: https://www.ars.usda.gov/northeast-area/beltsville-md-bhnrc/beltsville-human-nutrition-research-center/food-surveys- research-group.

We synthesised the Food and Agriculture Organization of the United Nations (FAO) food category classification guidelines and previous reports from similar literature using the adapted NHANSE food classification with the following DDS cumulative scoring methodology and food codes (first two digits) constructed on the basis of the five major food categories (18 subcategories in total):

| **Major food groups** | **Subgroups** | **Food code** | | | | | |
| --- | --- | --- | --- | --- | --- | --- | --- |
| **1 Grain products** | Whole grains | 56 | 57 | 58 |  |  |  |
|  | Non-whole grains | 50 | 51 | 52 | 53 | 54 | 55 |
| **2 Vegetables** | Dark green leafy | 72 |  |  |  |  |  |
|  | Vitamin A-rich | 73 | 74 |  |  |  |  |
|  | Starchy tubers | 71 |  |  |  |  |  |
|  | Others | 75 | 76 | 77 | 78 |  |  |
| **3 Fruits** | Citrus | 61 |  |  |  |  |  |
|  | Vitamin A-rich | 62 | 64 |  |  |  |  |
|  | Others | 63 | 67 |  |  |  |  |
| **4 Meat and protein alternatives** | Red meat or processed meat products | 20 | 21 | 22 | 23 | 27 | 28 |
|  | Poultry | 24 |  |  |  |  |  |
|  | Fish and seafood | 26 |  |  |  |  |  |
|  | Organ meat | 25 |  |  |  |  |  |
|  | Eggs | 31 | 32 | 33 |  |  |  |
|  | Legumes and nuts | 41 | 42 | 43 | 44 |  |  |
| **5 Dairy products** | Milks, milk drinks, yogurts | 11 |  |  |  |  |  |
|  | Concentrated Dairy Products | 12 |  |  |  |  |  |
|  | Solid Dairy Products (Milk desserts and sauces, andCheeses) | 13 | 14 |  |  |  |  |
